# Susceptibility Factors of Stomach for SARS-CoV-2 and Treatment Implication of Mucosal Protective Agent in COVID-19

**DOI:** 10.3389/fmed.2020.597967

**Published:** 2021-01-14

**Authors:** Min Zhang, Chao Feng, Xingchen Zhang, Shuofeng Hu, Yuan Zhang, Min Min, Bing Liu, Xiaomin Ying, Yan Liu

**Affiliations:** ^1^State Key Laboratory of Kidney Diseases, Department of Nephrology, The First Medical Center, National Clinical Research Center for Kidney Diseases, Chinese PLA Institute of Nephrology, Chinese PLA General Hospital, Beijing, China; ^2^Center for Computational Biology, Institute of Military Cognition and Brain Sciences, Academy of Military Medical Sciences, Beijing, China; ^3^Department of Pharmacy, The Central Hospital of Wuhan Affiliated to Tongji Medical College of Huazhong University of Science and Technology, Wuhan, China; ^4^Department of Integrated Traditional Chinese and Western Medicine, Union Hospital, Tongji Medical College, Huazhong University of Science and Technology, Wuhan, China; ^5^The Fifth Medical Center of Chinese PLA General Hospital, Beijing, China

**Keywords:** single-cell RNA sequencing, COVID-19, SARS-CoV-2, H pylori infection, intestinal metaplasia

## Abstract

**Objectives:** This work aims to study the gastrointestinal (GI) symptoms in severe acute respiratory syndrome coronavirus 2 (SARS-CoV-2)-infected patients and the susceptibility factors of the stomach for SARS-CoV-2.

**Materials and Methods:** We investigated the SARS-CoV-2 susceptibility by analyzing the expression distribution of viral entry-associated genes, *ACE2* and *TMPRSS2*, in single-cell RNA sequencing data derived from 12 gastric mucosa samples. We also analyzed the epidemiological, demographic, clinical, and laboratory data of 420 cases with SARS-CoV-2-caused coronavirus disease 2019 (COVID-19).

**Results:** *ACE2* and *TMPRSS2* are specifically expressed in enterocytes which are mainly from gastric mucosa samples with *Helicobacter pylori* (*H. pylori*) infection history and intestinal metaplasia (IM). A total of 420 patients were surveyed, of which 62 were with and 358 were without GI symptoms. There is a significant difference in average hospital stay (*p* < 0.001) and cost (*p* < 0.001) between the two groups. Among 23 hospitalized patients including seven with upper GI symptoms and 16 with lower GI symptoms, six (85.7%) and five (31.3%) had *H. pylori* infection history, respectively (*p* = 0.03). Of 18 hospitalized patients with initial upper GI symptoms, none of the eight patients with mucosal protective agent therapy (e.g., sucralfate suspension gel, hydrotalcite tablets) had diarrhea subsequently, whereas six out of 10 patients without mucosal protective agent therapy had diarrhea subsequently (*p* = 0.01).

**Conclusion:** IM and *H. pylori* infection history may be susceptibility factors of SARS-CoV-2, and the mucosal protective agent may be useful for the blockade of SARS-CoV-2 transmission from the stomach to the intestine.

## Introduction

The current SARS-CoV-2-caused coronavirus disease 2019 (COVID-19) pandemic is an ongoing global health crisis (1, 2). COVID-19 patients generally exhibited initial symptoms such as fever, fatigue, myalgia, dyspnea, and cough. Recent studies (3–5) showed that 20–50% patients had gastrointestinal symptoms as initial symptoms, and a large number of patients would have GI symptoms during hospitalization. SARS-CoV-2 RNA has also been detected in the patients' stools and will last a long time, suggesting that SARS-CoV-2 could be transmitted via the fecal–oral route (6, 7). SARS-CoV-2 transmission through the GI tract requires extensive attention.

The distribution of SARS-CoV-2 entry receptor may be highly associated with the route of infection, which is essential for understanding the pathogenesis mechanism (7–9). Recent studies (10, 11) reported that the viral host receptor ACE2 and the viral nucleocapsid were mainly in the cytoplasm of gastrointestinal epithelial cells. The scRNA-seq findings also uncovered that the SARS-CoV-2 entry receptor ACE2 and TMPRSS2 were specifically expressed in gastrointestinal epithelial cells such as enterocytes (7, 12). SARS-CoV-2 can invade the enterocytes and result in diarrhea. However, there are still a number of patients with non-diarrhea GI symptoms clinically, and it is still unknown whether these symptoms are due to stomach infection. As stomach is the upstream target organ in the fecal–oral route, a systematic survey of the distribution of SARS-CoV-2 entry receptor in the stomach and its susceptibility factors for SARS-CoV-2 infection will benefit our understanding of the mechanism of non-diarrhea GI symptoms and further guide effective prevention and treatment.

In this study, we aim to explore the susceptibility factors affecting gastrointestinal infections and the possible preventive or therapeutic measures using scRNA-seq data and the admission data of 420 laboratory-confirmed SARS-CoV-2 infection cases.

## Methods

### Analysis of Single-Cell and Bulk RNA Expression Matrices

The single-cell RNA expression matrices derived from 12 gastric mucosal samples were downloaded from the Gene Expression Omnibus [GEO, number GSE134520 (13)]. The bulk RNA sequencing expression matrices for human normal lung, colon, rectum esophagus, and stomach tissues were downloaded from the UCSC Xena website (https://xenabrowser.net/). We used the Seurat (14) package for scRNA-seq data analysis, including data integration, identification of highly variable genes, unsupervised graph-based clustering, differentially expressed genes, and dimension reduction using principal component analysis and Uniform Manifold Approximation and Projection. We also analyzed the expression of SARS-CoV-2 entry receptors in human normal lung, colon, rectum, and stomach tissues. We further performed Pearson correlation analysis between the expression levels of SARS-CoV-2 entry receptors with the average expression level of enterocyte markers (defined as enterocyte score) to validate the scRNA-seq findings.

### Study Design and Participants

In this retrospective, single-center study in Wuhan Central Hospital, we reviewed the admission data, including clinical records and laboratory test results, of 420 laboratory-confirmed SARS-CoV-2 infection cases from January 20 to April 30, 2020. According to the World Health Organization diagnostic guidelines and Chinese expert consensus of new coronavirus pneumonia prevention and treatment (15), the patients were divided into suspected cases and clinically diagnosed case. If the suspected case has the CT imaging features of COVID-19 pneumonia, it is classified as a clinically diagnosed case. The laboratory confirmed COVID-19 patients were diagnosed as positive for SARS-CoV-2 by real-time reverse transcription PCR. The patients with suspected and clinical diagnosis that have not been verified by laboratory examination are not included in this study.

The symptoms of COVID-19 are divided into four groups: mild, ordinary, serious, and critical groups according to the standard previously reported in Lin et al. (4), and the patients were further divided into non-severe (mild and ordinary) and severe (serious and critically) cases. The GI symptoms are divided into two parts: initial presentation group (IPG) and hospitalized presentation group (HPG) according to the occurrence time. The upper GI symptoms (UGIS) are defined with nausea/vomiting but without diarrhea, while diarrhea is defined as a lower GI symptom (LGIS). We also counted the occurrence rate of other non-specific GI symptoms such as anorexia and abdominal pain/abdominal discomfort. For patients with a co-occurrence of nausea/vomiting and diarrhea, we record the order in which the symptoms occur. We also investigated the admission examination and treatment of patients to record information such as gastric surgery history, upper gastrointestinal ulcer history, and *Helicobacter pylori* infection history. For those who had not been examined and recorded during the course of the disease, we followed up the information of *H. pylori* infection history and treatment within the last year.

### Statistical Analysis

All statistical tests were implemented with R statistical programming language (V.3.62). The continuous variables denoted as mean ± SD were compared by Wilcoxon test. The categorical data presented as percentage (%) were compared by χ^2^-test or Fisher's exact test. A two-sided *p* < 0.05 was considered as statistically significant.

## Results

### ScRNA-seq Analysis Reveals the Specific Expression of ACE2 and TMPRSS2 in Gastric Mucosa With *H. pylori* Infection and Intestinal Metaplasia

**Figure 1:**
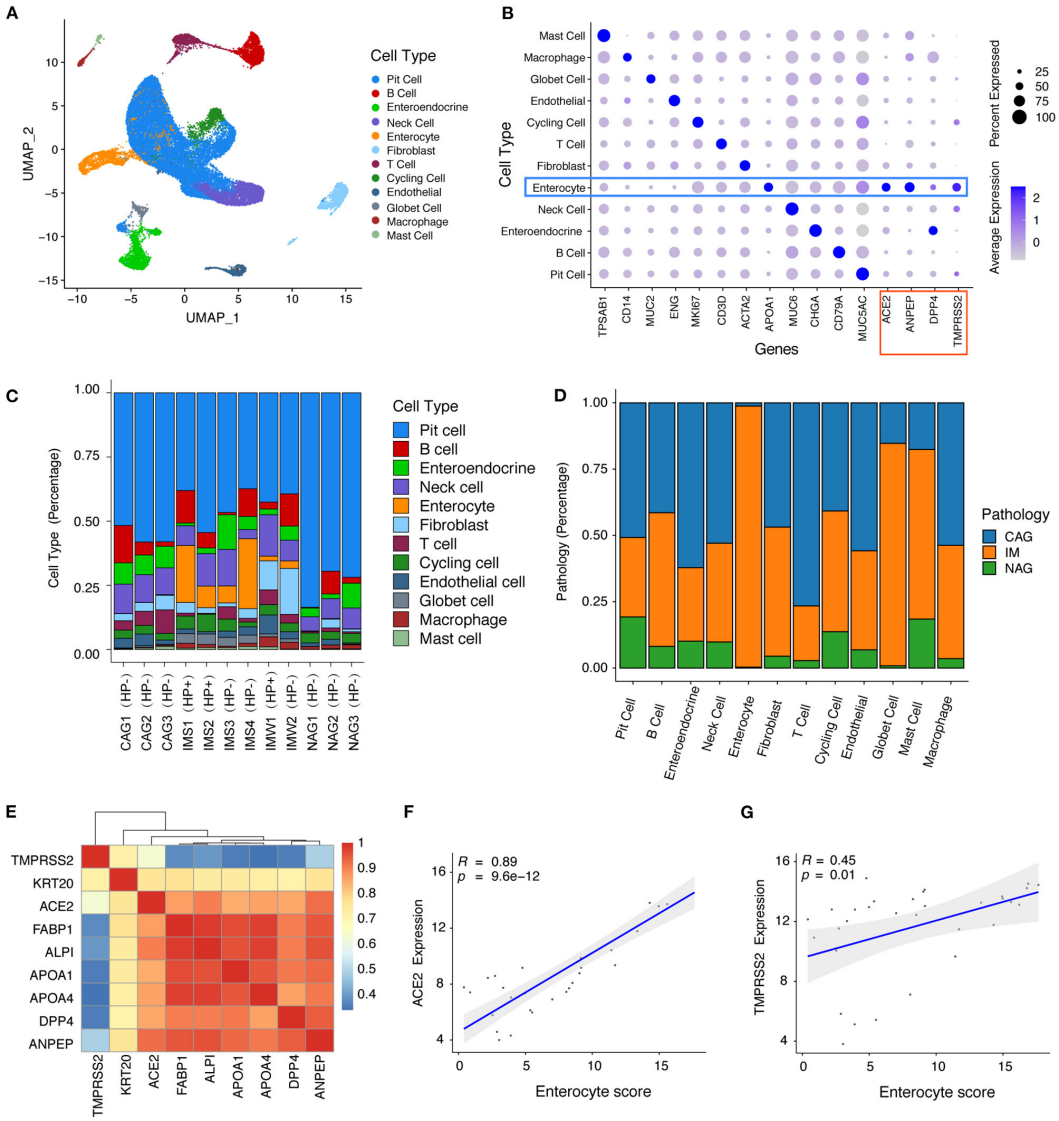
ScRNA-seq and bulk RNA-seq profiles reveal that ACE2 and TMPRSS2 were specifically expressed in gastric mucosa with *Helicobacter pylori* infection and intestinal metaplasia. **(A)** Unsupervised graph-based clustering revealed 12 cell types of 34,541 cells shown in Uniform Manifold Approximation and Projection plot. **(B)** Dot plot showing the expression of cell type markers and SARS-CoV-2 entry receptor ACE2 and TMPRSS2 and another two virus receptors (ANPEP and DPP4). **(C)** Bar plot showing the fraction of different cell types per sample. **(D)** Bar plot showing the fraction of different sample pathology per cell type. **(E)** Heat map showing the correlation coefficient between SARS-CoV-2 cell entry receptor ACE2 and TMPRSS2 with enterocyte genes. **(F-G)** Scatter plot showing the correlation coefficient of SARS-CoV-2 cell entry receptors **(F)** ACE2 and **(G)** TMPRSS2 with enterocyte gene score.

We analyzed 34,541 individual cells (Figure 1A) derived from 12 gastric mucosa samples (six intestinal metaplasia, IM; three non-atrophic gastritis, NAG; three chronic atrophic gastritis, CAG) of nine patients (two with and seven without *H. pylori* infection history). Unsupervised graph-based clustering revealed 12 cell types (Figure 1A), and the enterocytes specifically expressed SARS-CoV-2 entry receptor (9) ACE2 and TMPRSS2 and another two virus receptors (ANPEP receptor for HCoV-229E virus and DPP4 receptor for MERS-CoV virus) (Figure 1B). Interestingly, the vast majority of enterocytes were derived from gastric mucosa samples with *H. pylori* infection and IM (Figures 1C,D). The bulk RNA-seq profiles revealed that the expression levels of ACE2 and TMPRSS2 had a high correlation with the average expression levels of the enterocyte marker genes (Figures 1E,F), indicating that ACE2 and TMPRSS2 were specifically expressed in enterocytes. We further investigated the expression of ACE2 and TMPRSS2 in human lung, colon, rectum, and stomach. We found that ACE2 and TMPRSS2 have higher expression levels in intestinal-phenotype stomach (paired adjacent normal tissues of intestinal-phenotype gastric cancer) than those of gastric-phenotype stomach (paired adjacent normal tissues of diffuse-phenotype gastric cancer) (Supplementary Figures 1A,B). As we know, human IM stomachs are characterized by the emergence of intestine-specific cell types such as enterocytes (16), and *H. pylori* infection history is an important factor leading to IM (17). We speculate that SARS-Cov-2 can infect the stomach with *H. pylori* infection history and IM, thereby resulting in upper GI symptoms. These results also revealed that *H. pylori* infection history and IM might be susceptibility factors of SARS-Cov-2.

### A Systematic Survey of the Clinical Data of 420 Patients With COVID-19

A total of 420 COVID-19 patients (246 women and 174 men) were included in this study (Table 1). Most of the patients were non-severe (87.1%), and a few patients had smoking (2.1%) or alcoholism (0.07%) history. More than half of the patients had coexisting basic illnesses, and the most common illnesses are hypertension (27.1%), diabetes mellitus (12.3%), and cardio-cerebrovascular disease (8.8%) (Table 1). Among the 420 patients, 62 (14.8%) occurred with GI symptoms and 358 (85.2%) without GI symptoms. There was no statistically significant difference in the general demographics or clinical outcomes between the patients with and without GI symptoms. The patients with GI symptoms had a higher percentage of coexisting cardio-cerebrovascular disease than those without GI symptoms (*p* = 0.03). Interestingly, we found that the patients with GI symptoms had a significantly longer hospital stay (*p* < 0.001) and higher hospitalization costs (*p* < 0.001) than those without GI symptoms.

**Table 1:** The demographics, baseline features, and clinical outcomes of 420 patients infected with SARS-CoV-2.

	**All patients (*n* = 420)**	**Patients with gastrointestinal (GI) symptoms (*n* = 62)**	**Patients without GI symptoms (*n* = 358)**	***P*-value**
Age (year)	51.7 ± 17.5	53.0 ± 19.0	51.5 ± 17.3	0.62
Age groups				0.47
15–60	253 (60.2)	36 (58.1)	225 (62.8)	
>60	167 (39.8)	26 (41.9)	133 (37.2)	
Sex				0.10
Female	246 (58.6)	42 (67.7)	204 (57.0)	
Male	174 (41.4)	20 (32.3)	154 (43.0)	
Smoking history	9 (2.1)	0	9 (2.1)	0.37
Alcoholism history	2 (0.5)	0	2 (0.5)	1.00
Disease class				0.99
Non-severe	366 (87.1)	54 (87.1)	312 (87.2)	
Severe	54 (12.9)	8 (12.9)	46 (12.8)	
**Coexisting illness**				
Hypertension	114 (27.1)	11 (17.7)	103 (28.8)	0.07
Diabetes mellitus	52 (12.4)	7 (11.3)	45 (12.6)	0.78
Cardio-cerebrovascular disease	37 (8.8)	10 (16.1)	27 (7.5)	0.03
Malignant tumor	7 (1.7)	1 (1.6)	6 (1.7)	1.00
Chronic lung disease	18 (4.3)	4 (6.5)	14 (3.9)	0.57
Chronic kidney	9 (2.1)	1 (1.6)	8 (2.2)	1.00
Clinical outcome				0.63
Discharged	379 (90.0)	57 (91.9)	322 (90.0)	
Died	41 (10.0)	5 (8.1)	36 (10.0)	
Average hospital stay (day)	17.8 ± 9.4	24.2 ± 8.6	16.71 ± 9.1	7e-10
Average hospitalization cost (CNY)	21,658.0 ± 19,051.2	32,949.6 ± 22,542.1	19,702.5 ± 17,696.8	5e-7

The 62 patients with GI symptoms are classified into three groups: UGIS group (12 patients), LGIS group (30 patients), and non-specific GI symptoms group (20 patients). We compared the manifestations of patients in the UGIS group and the LGIS group (Table 2). No statistically significant differences are found in most general demographics, manifestations, or clinical outcomes between the UGIS and the LGIS groups except *H. pylori* infection and time from hospital admission to cardinal symptom onset. The average age of the patients with simple UGIS was 54.0 ± 17.0, higher than that of the patients with simple LGIS (50.1 ± 18.8), although the *p*-value is not significant (*p* = 0.57). We also investigated the presence of SARS-CoV-2 in feces for 36 hospitalized patients, including eight with UGIS and 28 with LGIS, of which three (37.5%) and 13 (46.4%) were positive for SARS-CoV-2, respectively (Table 2).

**Table 2:** Comparison of upper and lower gastrointestinal (GI) manifestations of 42 patients with SARS-CoV-2 infection.

	**Patients with upper GI symptoms (*n* = 12)**	**Patients with lower GI symptoms (*n* = 30)**	***P*-value**
Age (year)	54.0 ± 17.0	50.1 ± 18.8	0.57
Sex			1.00
Female	7/12 (58.4)	19/30 (63.3)	
Male	5/12 (41.7)	11/30 (36.7)	
Disease classification			0.67
Non-severe	9/12 (75.0)	25/30 (83.3)	
Severe	3/12 (25.0)	5/30 (16.7)	
**Coexisting illness**			
Hypertension	4/12 (33.3)	7/30 (23.3)	0.70
Diabetes mellitus	4/12 (33.3)	3/30 (10.0)	0.09
Cardio-cerebrovascular disease	4/12 (33.3)	6/30 (20.0)	0.43
Malignant tumor	0	1/30 (3.3)	1.00
Chronic lung disease	2/12 (16.7)	2/30 (6.7)	0.56
Chronic kidney disease	0	1/30 (3.3)	1.00
**Stomach diseases history**			
HP infection	6/7 (85.7)	5/16 (31.3)	0.03
Operation history	0	1/30 (3.3)	1.00
Ulcer	2/12 (16.7)	1/30 (3.3)	1.00
Died	1/12 (8.3)	2/30 (6.7)	1.00
Average hospital stay (day)	25.1 ± 9.0	23.47 ± 9.0	0.77
Average hospitalization cost (CNY)	32,113.4 ± 17,406.5	28,715.9 ± 20,360.5	0.40
**On initial presentation (IPG)**			
Cardinal symptoms	Nausea and vomiting (8)	Diarrhea (23)	
Concomitant symptoms	Inappetence (3) hematemesis (1)	Nausea (10) Inappetence (11)	
Duration of cardinal symptoms (day)	7.9 ± 4.6^*^	8.4 ± 3.2^#^	0.87
**During hospitalization (HPG)**			
Cardinal symptoms	Nausea and vomiting (4)	Diarrhea (7)	
Concomitant symptoms	Inappetence (3)	Inappetence (4)	
Time from hospital admission to symptom onset (day)	7.0 ± 2.9	2.4 ± 1.3	0.02
Duration of cardinal symptoms (day)	5.3 ± 3.6	3.4 ± 1.7	0.56
**Imaging examination**			
Not obvious	1/12 (8.3)	0	0.29
Patchy shadows involving both Lungs	9/12 (75.0)	26/30 (86.7)	0.39
Pulmonary consolidation/pleural effusion	2/12 (16.7)	4/30 (13.3)	1.00
**Laboratory examination**			
Fecal RNA test	3/8 (37.5)	13/28 (46.4)	0.70
WBC (<3.5 × 10^9^/L)	4/12 (33.3)	14/30 (46.7)	0.51
LYM (<1.1 × 10^9^/L)	6/12 (50.0)	17/30 (56.7)	0.74
NEUT (<1.8 × 10^9^/L)	1/12 (8.3)	6/30 (20.0)	0.65
MONO (>0.6 × 10^9^/L)	1/12 (8.3)	1/30 (3.3)	0.49
TBIL (>20 umol/L)	0	0	1.00
ALT (>40 U/L)	1/12 (8.3)	1/30 (3.3)	0.49
AST (>35 U/L)	1/12 (8.3)	4/30 (13.3)	1.00
CRP (>3 mg/L)	4/12 (33.3)	12/30 (40.0)	0.74

* *Duration of cardinal symptoms (upper GI symptoms) between IPG vs. HPG. p = 0.49*.

# *Duration of cardinal symptoms (lower GI symptoms) between IPG vs. HPG. p = 6.0e-4*.

Lymphopenia is the most common abnormal biochemical indicator in patients with COVID-19. In this study, 50% of patients in both UGIS and LGIS groups exhibited lymphopenia (Supplementary Table 1). We compared the lymphocyte counts of each patient at the time of hospitalization and recovery from discharge and found that nine (81.8%) and 24 (85.7%) had a lymphocyte count increase in the UGIS and LGIS groups, respectively. These results indicated that lymphocyte count is an important prognostic factor (18).

We also explored the association of *H. pylori* infection with GI symptoms for 23 hospitalized patients, including seven with UGIS and 16 with LGIS, of which six (85.7%) and five (31.3%) had *H. pylori* infection, respectively (*p* = 0.03, Table 2). These results indicate that *H. pylori* infection is associated with the presence of GI symptoms, especially for UGIS, which also supports our scRNA-seq findings that *H. pylori* infection might be a susceptibility factor of SARS-CoV-2.

According to the occurrence time of GI symptoms, patients from the UGIS and the LGIS groups were then further divided into two groups: IPG and HPG. We mainly focused on the difference of GI symptom duration between IPG and HPG and found that IPG had longer durations in both nausea/vomiting (7.9 ± 4.6 vs. 5.3 ± 3.6, *p* = 0.49) and diarrhea (8.4 ± 3.2 vs. 3.4 ± 1.7, *p* < 0.001) than HPG (Table 2). The results indicated that patients with timely clinical therapeutic intervention may help to accelerate the recovery process.

Based on this finding, we further investigated the correlation of timely clinical therapeutic intervention with clinical outcome. Among the 18 hospitalized patients with initial UGIS, eight with and 10 without mucosal protectant therapy (e.g., sucralfate suspension gel, hydrotalcite tablets), zero and six (60%) had subsequent diarrhea, respectively (*p* = 0.01, Table 3). We speculate that timely clinical therapeutic intervention may help to reduce virus load and to blockade SARS-Cov-2 transmission into the intestine.

**Table 3:** The clinical outcome of drug treatment involvement of COVID-19 patients with upper GI symptoms.

		**Upper GI symptoms (+) Subsequent diarrhea (+) (*n* = 6)**	**Upper GI symptoms (+) Subsequent diarrhea (–) (*n* = 12)**	***P*-value**
Mucosal protective agent^*^	Treated with	0	8	0.01
	Treated without	6	4	
Probiotics	Treated with	3	2	0.27
	Treated without	3	10	
Montmorillonite powder	Treated with	2	2	0.57
	Treated without	4	10	
Proton pump inhibitors	Treated with	6	12	1.00
	Treated without	0	0	
Prokinetic agents	Treated with	4	2	0.11
	Treated without	2	10	

* *Any drug that protects the mucosal lining of the stomach from acidic gastric juices, including sucralfate suspension gel, hydrotalcite tablets*.

## Discussion

Several recent studies have shown that SARS-Cov-2 needs to bind with ACE2 in order to invade human cells (10, 19, 20). The gastrointestinal epithelial cells express SARS-CoV-2 entry receptors (8); therefore, the GI tract (6, 7) may be a potential transmission route and target organ of SARS-CoV-2. Diarrhea is the most common GI symptom because SARS-CoV-2 could invade enterocytes. However, a number of patients had simple upper GI symptoms, i.e., nausea/vomiting but without diarrhea. Whether the stomach infection is related to UGIS and the susceptibility factors of the stomach for SARS-CoV-2 remain poorly investigated.

As we know, gastric IM is characterized by the emergence of intestine-specific cell types, including enterocytes. In China and many other countries, the incidence of gastric IM increases with age (21), and *H. pylori* infection is an important factor resulting in IM (16). In addition, we found that the stomach with *H. pylori* infection history and IM was enriched with enterocytes, and these cells specifically expressed SARS-CoV-2 entry receptor genes *ACE2* and *TMPRSS2*. The gastric mucosa with IM usually occurs alongside parietal cell loss and then leads to gastric juice PH elevation; thus, the SARS-CoV-2 virus is not inactivated by stomach acid (8). The normal gastric mucosa normally secrete gastric juice, and the PH is usually below 3. SARS-Cov-2 can be inactivated by gastric juice; thereby, it may not be infecting the normal stomach. Therefore, we speculate that the stomach with *H. pylori* infection history and IM may be susceptible to SARS-CoV-2.

Since it is unrealistic and difficult to check the stomach pathology, especially the IM status, in so many COVID-19 patients with GI symptoms, we conducted a systematic survey of the clinical data of 420 patients with COVID-19 to investigate the correlation of *H. pylori* infection history with GI symptoms. Interestingly, we found that most of the patients (six of seven) with UGIS and only five (31.25%) cases with LGIS had *H. pylori* infection history. This result, derived from 420 COVID-19 patients' clinical data, together with our findings on scRNA-seq data further provide evidence that *H. pylori* infection history and IM may be susceptibility factors of SARS-CoV-2 in the stomach.

In addition, our results revealed that the duration of GI symptoms in the HPG was shorter than that of IPG, suggesting the necessity of timely clinical therapeutic intervention. We further compared the clinical outcome of COVID-19 patients with UGIS with or without usage of mucosal protective agent. We found that the usage of mucosal protective agent reduced the occurrence of subsequent diarrhea. These results suggested that timely GI management, e.g., the usage of mucosal protective agent (e.g., sucralfate suspension gel, hydrotalcite tablets), will help to prevent further transmission from the stomach to the intestine through fecal–oral infection.

This study has limits since a small cohort of patients were enrolled; secondly, we did not perform a stomach biopsy examination in COVID-19 patients, especially the IM status. However, our scRNA-seq findings and the survey of 420 patients' data provided evidence that IM and *H. pylori* infection history may be susceptibility factors of SARS-CoV-2, and a mucosal protective agent may benefit to prevent further SARS-CoV-2 transmission.

## Data Availability Statement

The datasets presented in this study can be found in online repositories. The names of the repository/repositories and accession number(s) can be found at: https://www.ncbi.nlm.nih.gov/geo/, GSE134520.

## Ethics Statement

The studies involving human participants were reviewed and approved by the Central Hospital of Wuhan affiliated to Tongji Medical College of Huazhong University of Science and Technology (2020-112). The patients/participants provided their written informed consent to participate in this study.

## Author Contributions

MZ designed the experiments, analyzed the data, and wrote the manuscript. XZ, MM, and YZ collected the clinical data. CF and SH were responsible for data acquisition, analysis, and interpretation and writing of the manuscript. YL, XY, and BL were responsible for the study concept, design, and interpretation and revision of the manuscript. All the authors participated in the discussion.

## Conflict of Interest

The authors declare that the research was conducted in the absence of any commercial or financial relationships that could be construed as a potential conflict of interest.

## Supplementary Material

Supplementary Figure 1 Bulk RNA-seq profiles showing the expression of ACE2 **(A)** and TMPRSS2 **(B)** in normal human lung, colon, rectum, and stomach. NOS, not otherwise specified.

Supplementary Table 1 The clinical laboratory results alteration trend at the time of hospitalization and recovery from discharge.
