# Supplementary figures and images for: Susceptibility Factors of Stomach for SARS-CoV-2 and Treatment Implication of Mucosal Protective Agent in COVID-19
Source: Front Med (Lausanne). 2021 Jan 14;7:597967. doi: 10.3389/fmed.2020.597967 (PMC7840564; doi:10.3389/fmed.2020.597967)

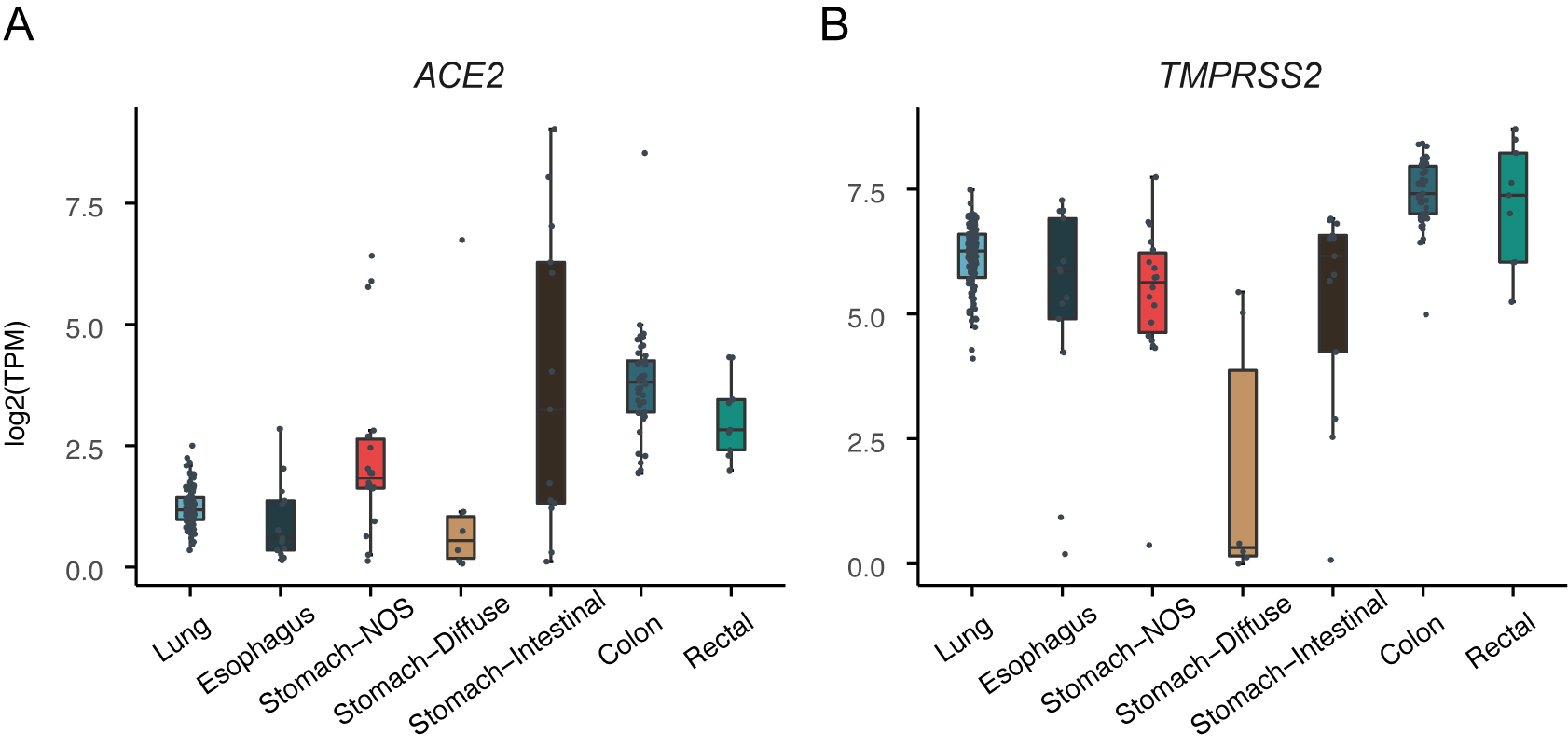

Supplement: Supplementary Figure 1 — Bulk RNA-seq profiles showing the expression of ACE2 (A) and TMPRSS2 (B) in normal human lung, colon, rectum, and stomach. NOS, not otherwise specified. [file Image_1.TIF]
